# Drone-based investigation of natural restoration of vegetation in the water level fluctuation zone of cascade reservoirs in Jinsha River

**DOI:** 10.1038/s41598-022-14578-z

**Published:** 2022-07-28

**Authors:** Weiwei Jiang, Wentao Li, Jianguo Zhou, Pengcheng Wang, Henglin Xiao

**Affiliations:** School of Civil Engineering, Architecture and Environment, Hubei University of Technology, Wuhan, Hubei Province People’s Republic of China

**Keywords:** Riparian ecology, Wetlands ecology

## Abstract

The reservoir water level fluctuation zone (WLFZ) is a new and fragile ecosystem that is gaining attention with the construction of large and medium-sized hydropower plants. Compared to the natural riparian zone, it has a greater drop in water level, longer inundation time, more intense impact from alternating wet and dry conditions, and a wider impact on ecological security. The Jinsha River basin is located in the upper reaches of the Yangtze River in China, and several world-class large-scale hydropower projects with dam heights over 100 m have been built, forming a large area of reservoir WLFZ, however, due to the short time since their construction, there are few related studies. In this paper, fixed sample plots were set up in the typical WLFZs of each large reservoir in the Jinsha River basin. In response to the problem of the precipitous terrain and poor accessibility of the Jinsha River basin, a combination of small UAV surveys and field research in July 2020 was used to draw vegetation cover maps and extract topographic data for each site, and quantitatively analyse the community composition, dominant species types, area, coverage, spatial distribution patterns and environmental factors of tolerant vegetation using spatial superposition analysis, neural network models, landscape pattern indices and typical correlation analysis. The results showed that the original drought-tolerant vegetation in the arid river valley WLFZ has evolved into amphibious herbaceous vegetation, with trees and shrubs disappearing and species composition tending to be simpler. 44 species of plants, mainly in the Asteraceae and Gramineae families, were extant, 61% of which were also reported in the Three Gorges Reservoir WLFZ. The water level variation showed convergence in the natural screening process of suitable species in the WLFZ. Moreover, even in the dry valley WLFZs, flood stress showed a more significant filtering effect on vegetation species than drought stress. The vegetation in the WLFZ showed an obvious band-like aggregated distribution along the water level elevation gradient, and the vegetation coverage along the flooding gradient is as follows: upper part of the WLFZ >> middle part > lower part, and mainly concentrated in the gentle area with slope less than 35°. Flooding stress, drought stress and soil substrate deficiency were the main limiting factors for vegetation recovery in the WLFZ. The vegetation restoration of the WLFZ should be adapted to local conditions, and the dominant role of native species should be emphasized. At the early stage of the restoration of the WLFZ, native species should be selected for artificial planting to accelerate the formation of vegetation cover, and gradually advance downwards along the gradient of water level elevation, while for areas of the WLFZ with slopes greater than 35° and large topographic relief, biological engineering measures should be used to help plant establishment, and after a certain stable cover has been formed, natural restoration should be the main focus.

## Introduction

Dams are one of the most effective means for human beings to exploit water resources. They are a major infrastructure that affects the people's livelihood and play an important role in ensuring the safety of people's lives and property and the sustainable development of the country's economy and society. According to the International Commission on Large Dams (ICOLD)^1^, as of April 2020, there are 58,713 dams (dams greater than 15 m in height, or dams greater than 5 m in height and with a capacity greater than 3 million m^3^) worldwide. Reservoir water level fluctuation zone (WLFZ) is a new fragile ecosystem gradually attracted attention with the construction of large and medium-sized hydropower stations. It refers to the drawdown area formed between the highest water level and the lowest water level due to seasonal water level drawdown and periodic water storage^2^. Initially, countries such as North America, Europe, Australia, Japan, and South Africa referred to the transitional area of terrestrial and aquatic ecosystems as “riparian zones”. In the 1970s, the importance of riparian ecosystems was discussed by scholars^3^. In recent years, the over exploitation of riparian ecosystems by humans, driven by economic interests, has led to a reduction in biodiversity, increased non-point source pollution and a poor ecological environment in the riparian zone, and increasing attention is being paid to restoring riparian ecosystems.

Riparian zone was first considered to be complex collections of plants and other organic matter in the environment adjacent to water, a transition zone between the aquatic environment and the mountainous terrain, rather than being considered as ecosystems. The WLFZ draws on the definition of riparian zone, stating that the WLFZ is an area of alternating terrestrial and aquatic ecosystems and is a separate ecosystem. Compared with the riparian zone, the WLFZ has longer flooding time, larger water level drop and obvious flooding gradient, and its water level fluctuation has a more severe impact on vegetation. Especially for the reservoirs with the strategy of “clear storage and muddy discharge” , their WLFZs usually show "winter water and summer land”, and the rhythm of water level rise and fall is completely opposite compared with the natural riparian zone before the construction of reservoirs^4^. Taking the Three Gorges Reservoir WLFZ, located in the middle and lower reaches of the Yangtze River basin in China, as a representative, a lot of research^5–9^ has been conducted on flora and fauna, microorganisms, soil, heavy metals, and slope stability in the WLFZ, and a series of progress has been made in curbing water and land pollution, soil erosion, environmental and geological disasters, protecting biodiversity and ecosystem reconstruction. However, there are fewer studies related to reservoir WLFZs in the Jinsha River basin.

The Jinsha River, in the upper reaches of the Yangtze River in China, originates from the Tongtian River in Qinghai and flows through the Qinghai-Tibet Plateau, the Yunnan-Guizhou Plateau and the western edge of the Sichuan Basin, with a total length of about 3500 km, a natural drop of about 5100 m, and a basin area of about 473,200 square kilometers^10^. With its steep slope and rapid flow, abundant and stable water volume, large and concentrated drop, Jinsha River is rich in hydropower resources, and the degree of enrichment of its cascade hydropower projects is among the highest in the world^11–13^, with a number of large hydropower projects with dam heights exceeding 100 m. With the cascade development of the basin's hydropower, a water level fluctuation zones (WLFZ) with periodic alternation of land and water has been formed around the reservoir bank^14,15^. As a result of the reservoir's operation strategy of "clear storage and muddy discharge", the water level in the WLFZ changes in the opposite direction to the natural flooding pattern, showing "winter water and summer land", with the water level drop being several to dozens of times that of the original natural riparian zone, and the duration of flooding lasting for several months. Due to the long flooding time and large fluctuation range, the original vegetation in the WLFZ almost disappeared^16,17^. Vegetation can intercept large amounts of sediment from land bank erosion, prevent bank failure due to river erosion, and plays a key role in nutrient cycling and filtering non-point source pollutants^18–20^. Therefore, the extinction of original vegetation in the WLFZ seriously threatens the sustainable utilization of water conservancy and hydropower projects and the benign cycle of ecological environment in the reservoir area.

As the main body of ecosystem functions in the WLFZs and riparian zones^21^, the value of vegetation has always attracted the attention of scholars at home and abroad. How to slow down and prevent the degradation and shrinkage of vegetation in the WLFZ and riparian zone, and restore and reconstruct the damaged ecosystem has become a hot research topic nowadays. Using a combination of pioneer species and bioengineering measures, Zhang et al^22^. designed a restoration plant community structure optimization allocation model for diving degraded riparian zones in Anhui province. The restored riparian zone ecosystems showed increased biodiversity and stability, and improved soil structure and nutrient conditions. Shley et al^23^ applied a specific methodology for revegetation in Australia, investigating residual vegetation and considering flood disturbance, vegetation allocation, vegetation succession, planting density, planting techniques, native vegetation regeneration, and appropriate ecosystem management. A great deal of research and practical work has been carried out around the ecological restoration of the Three Gorges Reservoir WLFZ in the middle and lower reaches of the Yangtze River^24–26^. However, for the Jinsha River reservoirs located in the upper reaches of the Yangtze River, there are fewer related studies due to their remote location, complex topography, and the fact that many reservoirs have just been built. The climate, topography, soil conditions and native vegetation types, etc. of the Jinsha River reservoir WLFZs differ greatly from those of the Three Gorges reservoir WLFZ, especially as many of the reservoir WLFZs are located in typically arid river valley areas, where water-heat conflicts are prominent and the natural recovery status of the WLFZs after experiencing high levels of flooding is unknown. There is a great deal of uncertainty whether the research results and practical experience related to vegetation in the reservoir WLFZ, such as in the Three Gorges reservoir area, can be adapted to dry river valley terrace reservoirs. It is particularly necessary and urgent to conduct a basic survey of the vegetation in the WLFZs of Jinsha River Reservoirs to clarify the current status of the vegetation in the WLFZs, including the composition of existing species, coverage, spatial distribution pattern and its influencing factors, etc. Existing species are those that have survived natural selection and are well adapted to the local environment, and are therefore often the recommended choice of plant for restoration of WLFZ^27–29^. The area proportions and spatial distribution patterns of the various vegetation cover classes can provide a natural reference for species ratios and spatial allocations of restoration plants, respectively. The analysis of the factors influencing the restoration of vegetation in the WLFZ can provide theoretical guidance for the scientific protection and restoration of the ecological environment of the WLFZ.

The Jinsha River reservoir area is located in high mountain valley terrain, making many areas inaccessible and traditional ground survey methods are greatly limited to obtain continuous observations over large areas of space. Satellite remote sensing and airborne remote sensing are not limited by topography and can obtain spatially continuous data, but the spatial resolution of both cannot identify low herbaceous vegetation in the WLFZ and are vulnerable to cloud cover above the reservoir. In contrast, UAVs are mobile, flexible, economical, and able to fly at low altitudes under clouds, allowing sensors to acquire high enough spatial resolution from the right altitude to meet the needs of fine vegetation surveys^30–32^. Therefore, this paper used a light UAV as a survey tool, combined with field research, to reveal the current situation and environmental impact factors of the natural restoration of vegetation in the Jinsha River reservoir WLFZs in an intuitive and quantitative way, providing a scientific basis for the ecological restoration of the reservoir WLFZ.

## Materials and methods

### Study area

**Figure 1:**
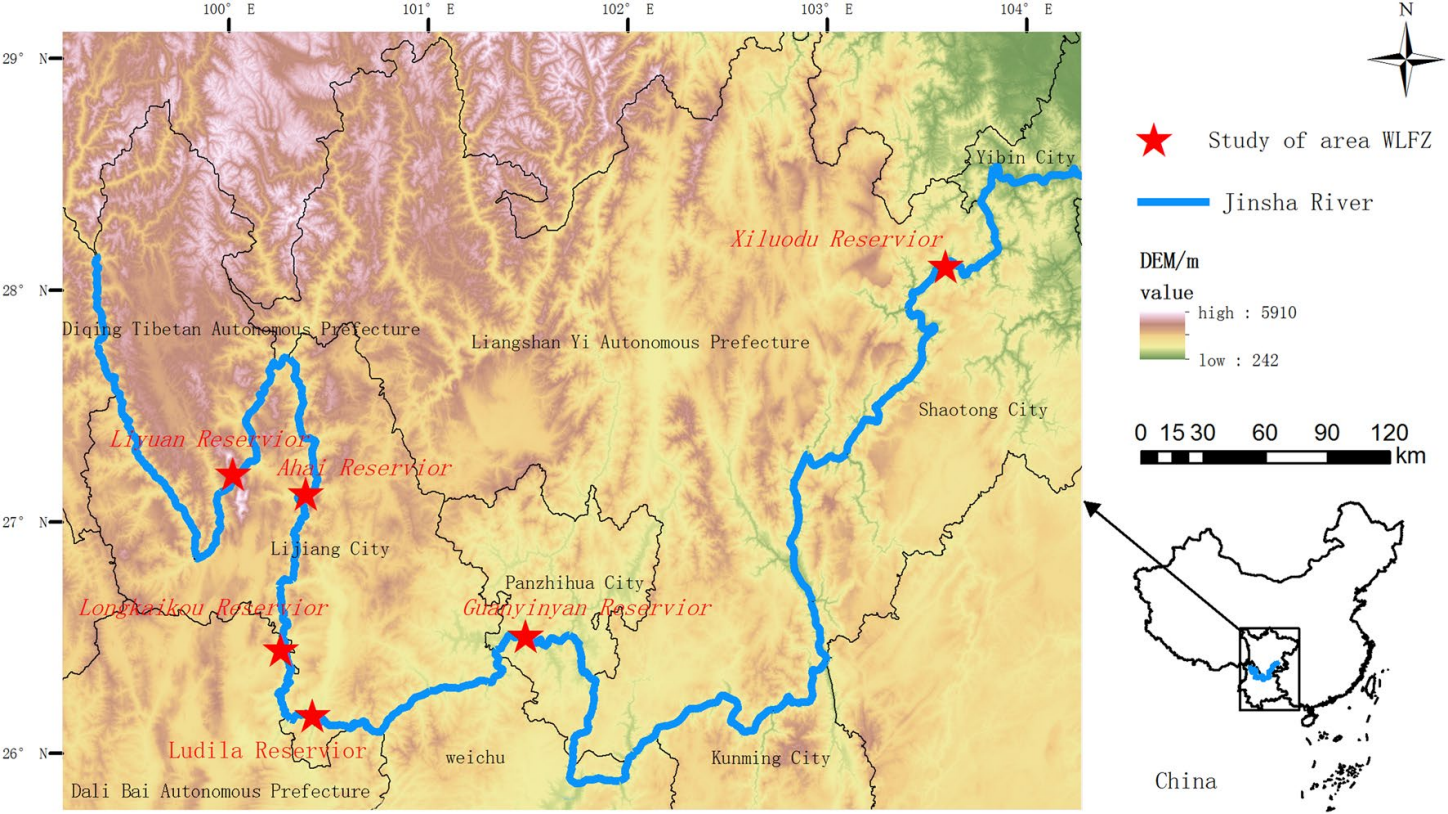
Location map for WLFZ study area (Drawn with ArcGIS 10.5 software, and the URL is: https://www.esri.com/en-us/home. The SRTM 90 m DEM map contained is provided by Geospatial Data Cloud site, Computer Network Information Center, Chinese Academy of Sciences. http://www.gscloud.cn).

In Jiansha River, Liyuan Reservoir, Ahai Reservoir, Longkaikou Reservoir, Ludila Reservoir, Guanyinyan Reservoir and Xiluodu Reservoir are six cascade reservoirs that have formed a large area of WLFZ. Fixed observation sites were set in the typical degradation WLFZ of these reservoirs as shown in Fig. 1 The initial storage time of each reservoir were varied from December 2011 to November 2014, so the original vegetation in the WLFZ of each study area was flooded more than 5 years ago. Since the first impoundment of these reservoirs, the water level starts to drop in February every year, and reaches a minimum in May to keep the water level low, until August or September when the water level gradually starts to rise again, forming a seasonal WLFZ that is periodically exposed and opposite to the natural flooding and dring pattern of Jinsha River. The Jinsha River basin has distinct wet and dry seasons, and in its natural state, the dry season is usually from November to May each year. Except for the Xiluodu Reservoir which is in a warm and wet valley, the other five study areas are in dry-hot and dry-warm valleys^33,34^ with abundant sunshine and large evaporation, average annual temperature of 20–23 °C, annual precipitation of 600–800 mm, and annual evaporation 3–6 times of annual precipitation.

### Field data acquisition

**Figure 2:**
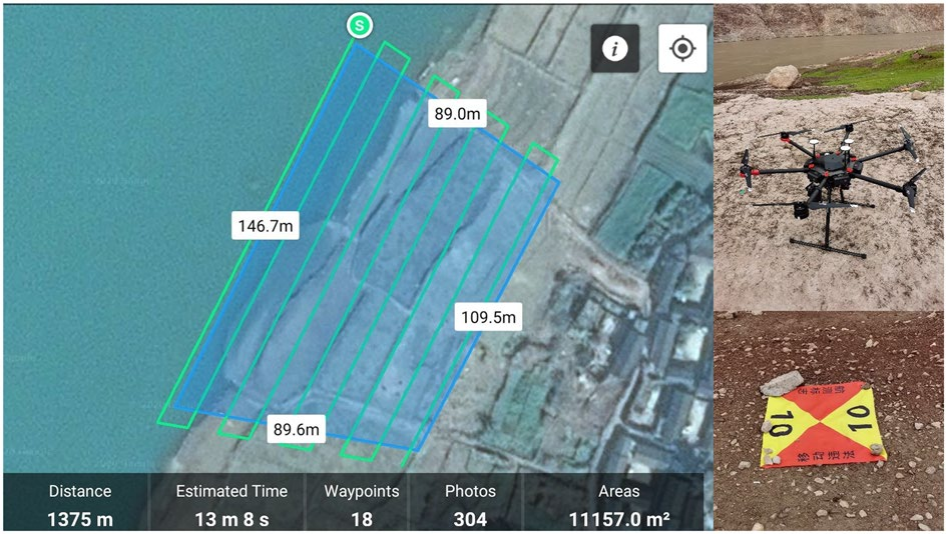
Route planning, UAV and control point mark (Drawn with WPS Office, and the URL is https://www.wps.cn/).

A DJI M600 PRO six-rotor UAV with 12 million pixels digital camera was used as the data acquisition platform, and DJI Pilot software was used for flight and photo control (Fig. 2). For each study area, the course overlap of UAV was 85%, the lateral overlap was 70–75%, and the flight speed was less than 2.5 m/s. Considering the undulating topography of the experimental area, the average altitude was 24 m (range = 18–30 m). Before flying, 8–10 image control points were evenly arranged in each study area, and the longitude, latitude and elevation (all elevations in this paper refer to ellipsoidal heights) of the control points were measured by the Continuously Operating Reference Stations (CORS) using Kelida K9 series GPS (static plane accuracy: ± 3 mm + 1 ppm, static elevation accuracy: ± 5 mm + 1 ppm).

Following the completion of aerial photography of each study area, on-site investigation was carried out to record, through photographs and text, the names of plant species in the WLFZ, their heights and distribution, the upper boundary of the inundation, etc. The thickness of the soil layer was also measured from 0 to 30 cm below ground. And three sampling points were evenly spaced in the upper, middle and lower part of the WLFZ. The Ph, N, P and K contents were measured at each point from the 0–10 cm, 10–20 cm and 20–30 cm layers below ground respectively by a hand-held rapid testing platform. The elevation of each sampling point was measured based on GPS-CORS.

### Vegetation mapping of WLFZ

The UAV aerial flight images and image control points were imported into Pix4Dmapper software to process orthophoto image initialization, point cloud-grid generation, orthophoto image and digital surface model (DSM) generation^35^ in the study area. According to the Pix4Dmapper report, the resolution of orthophoto images in each study area is less than 1.2 cm.

Taking eCognition as the algorithm platform, a set of unique processing flow (Process tree) was designed to classify the orthophoto images in each study area. First, the fractal net evolution approach (FNEA) algorithm^36^ was used for multi-scale segmentation of the image (scale parameter = 10, shape parameter = 0.4, compactness parameter = 0.8). Then, the features of the segmented objects were extracted (Table 1), and the vegetation in the WLFZ was classified as the combination of rule-based classification and visual interpretation.

**Table 1:** Features extracted for classification.

Name	Abbreviation	Formula	Reference
Red	R	R	–
Green	G	G	–
Blue	B	B	–
DSM	DSM	DSM	–
Green–Red	Rg-Rr	Rg-Rr	–
Green–Blue	Rg-Rb	Rg-Rb	–
Excess green	EXG	2Rg-Rr-Rb	^37^
Excess green minus excess red index	EXGR	EXG-1.4Rr-Rg	^38^
Vegetation index	VEG	Rg/Rr0.67Rb0.33	^39^
Color Index of Vegetation	CIVE	0.441Rr-0.881Rg + 0.385Rb + 18.78745	^40^
Water index	WI	(Rg − Rb)/(Rr − Rg)	^41^
Combination	COM	0.25EXG + 0.3EXGR + 0.33CIVE + 0.12VEG	^42^
Combination 2	COM2	0.36EXG + 0.47CIVE + 0.17VEG	^43^

With the first layer being the extraction region of the WLFZ, a rule (Formula 1) was established using the DSM data, in which $h_{1}$ is the elevation of the water surface and $h_{2}$ is the elevation of the upper boundary of the WLFZ. The images were divided into three types: the WLFZ, the water body and the area above the WLFZ. Misclassification caused by floating objects on the water surface was edited and corrected manually by visual interpretation.

1 $$\left\{ \begin{array}{l} class1 \, (WLFZ):\{ H_{dsm}\} \,{\text{gt}}\,h_{1}\, \&\, \{H_{dsm}\} \, \text{lt}\,h_{2} \hfill \\ class2 \, (water): \, \{H_{dsm} \} \, le \, h_{1} \hfill \\ class3 \, (above \, area): \{H_{dsm}\} \, ge \, h_{2} \end{array} \right.$$

The second layer is the distinction between vegetation and non-vegetation. According to the specific conditions of the image vegetation in each experimental area, a rule set was constructed by using one or a combination of features (Table 1), as shown in Formula 2, in which $x_{i}$, $y_{i}$ represents the upper and lower threshold of the rule respectively, and distinguishes vegetation from non-vegetation from the category of WLFZ combined with visual interpretation, that is, the WLFZ was further divided into two types: vegetation and non-vegetation.

2 $$\begin{aligned}class \, (WLFZ) \, \left\{ \begin{array}{l} class1 \, (vegetation): \{ F_{i} \} \, gt \, x_{i} \, \&\, \{ F_{i} \} \, lt \, y_{i} , i = 1,2,\ldots,n; \hfill \\ \qquad\quad F_{i} \in (R_{r} , R_{g} , R_{b} ,\ldots , \, BGRI) \hfill \\ class2 \, (non - vegetation): otherwise; \hfill \\ \end{array} \right. \end{aligned}$$

Layer 3-n is the distinction of vegetation types. The rule sets were constructed separately for each dominant species in each study area, and combined with visual interpretation, the dominant species categories were further extracted in a hierarchical manner from the vegetation classes classified in the previous step, and the non-dominant species in the experimental area were unified into Other-Veg class. The rule sets of classification for each study area is shown in Table 2, where the extraction of dominant species can be done using fewer rules, relying overwhelmingly on manual visual interpretation.

**Table 2:** The rule sets of classification for each study area.

Study area	Category	Rule description
Liyuan	Water	DSM < 1572.68
	Non-WLFZ	DSM > 1584.68
	WLFZ	DSM ≥ 1572.68 and DSM ≤ 1584.68
	Vegetation	G-B ≥ 0 and G-R ≤ 0
Ahai	Water	DSM < 1460.22
	Non-WLFZ	DSM > 1470.59
	WLFZ	DSM ≥ 1460.22 and DSM ≤ 1470.59
	Vegetation	COM2 ≥ CIVE
	*C. dactylon*	G-B ≤ 20 and G-R ≤ 20
Longkaikou	Water	DSM < 1254.52
	Non-WLFZ	DSM > 1264.7
	WLFZ	DSM ≥ 1254.52 and DSM ≤ 1264.7
	Vegetation	G-R ≥ 0 and G-B ≤ 0
	*C. dactylon*	CIVE ≥ − 15
Guanyinyan	Water	DSM < 1086
	Non-WLFZ	DSM > 1096
	WLFZ	DSM ≥ 1086 and DSM ≤ 1096
	Vegetation	G-R ≥ 0 and G-B ≥ 0
	*X. sibiricum*	DSM ≥ 1091.5, EXG ≥ 50, G-B ≥ 28 and G-R ≥ 28
Xiluodu	Vegetation	G-R ≥ 0 and G-B ≥ 0
	*X. sibiricum*	CIVE ≤ − 12
	*C. dactylon*	EXG ≤ 52 and WI > − 1.18
	*S. viridis*	COM ≤ − 125 and EXGR ≤ − 432

Classification results were evaluated by user accuracy $p_{ui}$ and producer accuracy $p_{Ai}$, both were calculated based on ground truth reference maps collected by random intensive sampling, as shown in Formula 3 and Formula 4, in which $p_{ii}$ represents the number of correctly classified pixels of category $i$; $p_{ci}$ represents the number of all pixels classified into category $i$; $p_{ri}$ represents the total number of pixels of category $i$ in the ground truth reference map. User accuracy reflects misclassification, and producer accuracy reflects missing classification.

3 $$p_{ui} = p_{ii} /p_{ci}$$

4 $$p_{Ai} = p_{ii} /p_{ri}$$

### Analysis of spatial distribution pattern and influence of topographical factors

Using ArcGIS software and Fragstats 4.2 software, the landscape pattern index was calculated based on the vegetation classification map^44^ to analyze the spatial pattern of vegetation in the WLFZ, where the landscape pattern index was selected from three aspects: area, shape and aggregation, landscape area (CA), maximum patch index (LPI), shape index (SHAPE), perimeter area fractional dimension index (PAFRAC), mean proximity index (PROX) and mean nearest neighbor distance (ENN).

Since the landcover in the WLFZ were mainly bare soil, low herbaceous vegetation and stones with no tall features, six common terrain factors including elevation, slope, aspect, surface relief, surface roughness and topographic wetness index^45^ were calculated and extracted based on the digital surface model of the study area generated in “Vegetation mapping of WLFZ” section ßßusing ArcGIS software^46,47^. Then the topographic factors were spatially overlaid with the vegetation classification map of the WLFZ based on ArcGIS software, and the segmental statistics of each topographic factor were performed separately to quantitatively obtain the spatial distribution characteristics of vegetation along the terrain.

**Figure 3:**
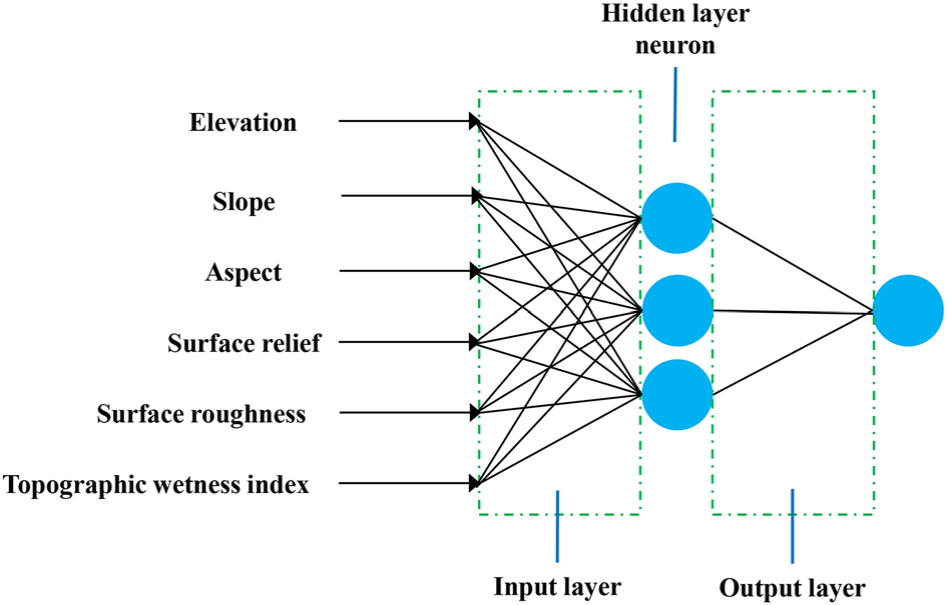
Schematic diagram of neural network model (Drawn with WPS Office, and the URL is https://www.wps.cn/).

After that, sample data of vegetation distribution were collected from the vegetation classification map of the study area with each terrain factor data, to construct a neural network multilayer perceptron model (Fig. 3), so as to comprehensively analyze the influence of each terrain factor on vegetation distribution. Among them, the topographic factors were used as independent variables and vegetation as dependent variables, 60% of the samples were used for training and 40% for testing.

**Figure 4:**
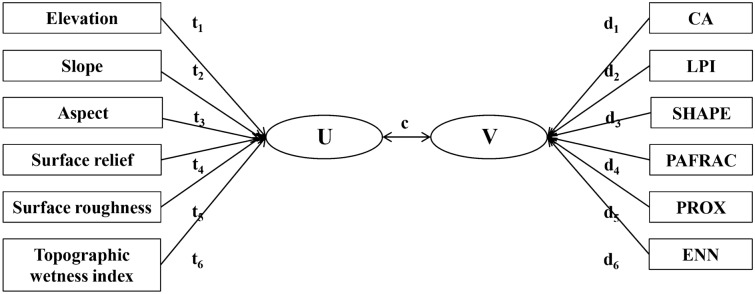
Diagram of canonical correlation analysis (Drawn with WPS Office, and the URL is https://www.wps.cn/).

To further analyze the influence of each topographic factor on the vegetation landscape pattern, the calculated spatial distribution map of the landscape pattern index was subjected to a typical correlation analysis with the topographic factor using spss25 software^48^ (Fig. 4), and the two sets of variables were the topographic factor and the landscape pattern index, where (t1,t2,t3,t4,t5,t6) were the topographic factor coefficients, (d1,d2,d3,d4,d5,d6) were the landscape pattern index coefficients, and c was the correlation coefficient.

## Results

### Species composition of vegetation in the WLFZ

In this survey, a total of 44 species in 43 genera of 21 families of vascular plants were found and confirmed in the reservoir WLFZ of the Jinsha River basin, among which, 13 genera and 13 species of *Compositae*, 4 genera and 4 species of *Gramineae*, 3 genera and 3 species of *Amaranthaceae*, 2 genera and 2 species of *Verbenaceae*, *Labiatae*, *Umbelliferae*, *Cruciferae* and *Convolvulaceae*, 1 genus and 2 species of *Polygonaceae*, and the remaining 12 families were all single genera. *Compositae* had the highest number of species, followed by *Gramineae* and *Amaranthaceae*, accounting for 29.55%, 9.09% and 6.82% of the total number of species in this survey, respectively, which are the main dominant families in the region.

According to the life type classification system of the Flora of China, the plants in the WLFZ of this survey can be classified into five life types: annual herbs, perennial herbs, annual or biennial herbs, annual or perennial herbs, and biennial herbs. The community is overwhelmingly dominated by annuals with a high proportion of 54.55%, followed by perennials with 34.09% and the rest of all life types with a total of 11.36%.The higher number of annual plants indicates that the environmental conditions in the WLFZ are harsher after inundation by water storage, and plants that can complete their entire life cycle in a short period of time after receding water are more likely to survive compared to plants that take a long time to complete their entire life cycle.

The vegetation types in each study area of the WLFZ are shown in Table 3, among which 17 species, including S. subulatum, E. humifusa, C. bonariensis, V. officinalis, O. biennis, S. plebeia, U. fissa, B. juncea, S. orientalis, D. repens, A. lividus, T. mongolicum, G. parviflora, P. praeruptorum, P. hys-terophorus, D. stramonium and Ph. Nil, are newly discovered species in the reservoir WLFZ, which are rarely reported in other reservoir WLFZ studies so far. Among the study areas, the Longkou study area was the richest in vegetation types, with the most families, species and life types among all study areas, and the number of perennial herb species was comparable to that of annual herb species, while all other study areas were mainly dominated by annual herbs. The vegetation composition of the remaining study areas averaged 6–8 families and 11–12 species, except for the Ludila study area with no plants growing and the Liyuan study area with only 5 families and 5 species. In general, each study area was dominated by *Compositae* and *Gramineae*.

**Table 3:** Vegetation composition in each study area.

Study area	Species	Life type/species number	Families	
			Number	Dominant/proportion
Liyuan	*S. subulatum, Ch. ambrosioides, E. indica, V. peregrine, E. humifusa*^*a*^	Annual herb/4	5	–
		Annual or perennial herb/1		
Ahai	*C. dactylon, P. lapathifolium, S. subulatum, Ch. ambrosioides, P. plebeium, R. indica, E. indica, A. sessilis, V. peregrine, C. rotundus, A. argyi*	Annual herb/6	8	Gramineae, Compositae, Polygonaceae, 18.18% each
		Perennial herb/4		
		Annual or perennial herb/1		
Longkaikou	*C. dactylon, O. corniculata,Ch. ambrosioides, C. bonariensis, P. lapathifolium, V. officinalis, S. subulatum, G. odoratum, B. pilosa, A. sessilis, O. biennis, P. asiatica, M. Canadensis, S. plebeia, U. fissa, G. affine, H. sibthorpioides, B. juncea, S. orientalis, D. micrantha, A. blitum, T. mongolicum, E. Canadensis, R. indica, G. parviflora, A. conyzoides, M. coromandelianum*	Annual herb/11	16	Compositae 33.33%
		Perennial herb/11		
		Annual or perennial herb/2		
		Annual or biennial herb/2		
		Biennial herb/1		
Ludila	*Null*	–	–	–
Guanyinyan	*A. sessilis, X. sibiricum, C. dactylon, E. indica, D. sanguinalis, Ph. nodiflora, P. lapathifolium, S. subulatum, P. oleracea, C. rotundus, E. prostrata, P. praeruptorum*	Annual herb/7	8	Compositae, Gramineae, 25% each
		Perennial herb/5		
Xiluodu	*C. dactylon, S. viridis, E. indica, P. hysterophorus, X. sibiricum, A. conyzoides, P. oleracea, D. sanguinalis, C. argentea, D. stramonium, Ph. nil*	Annual herb/10	6	Gramineae 6.36%, Compositae 27.27%
		Perennial herb/1		

### Vegetation area, coverage, and percentage of the WLFZ

**Figure 5:**
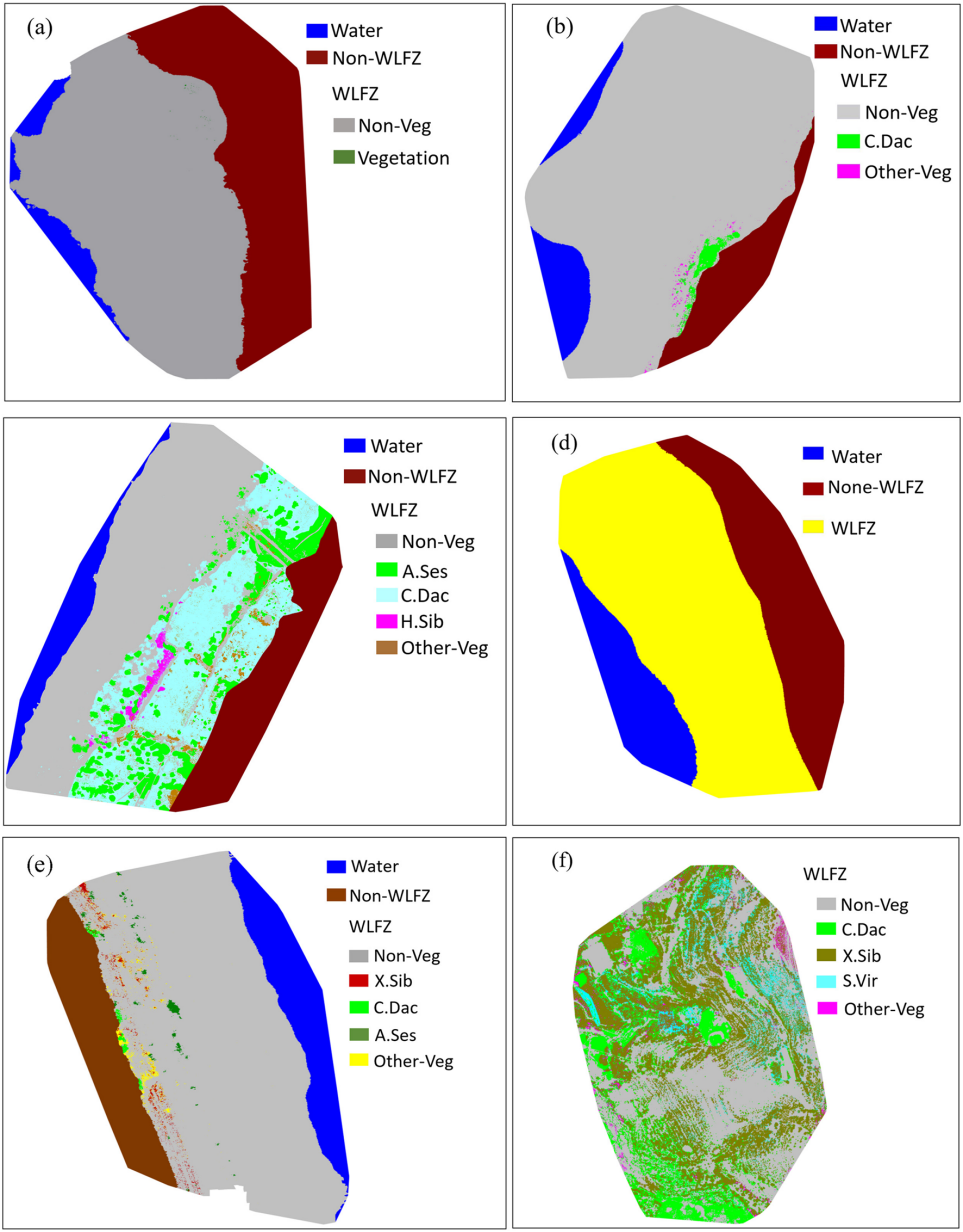
The results of vegetation classification in the WLFZ of each study area. (**a**) Liyuan, (**b**) Ahai (**c**) Longkaikou, (**d**) Ludila, (**e**) Guanyinyan, (**f**) Xiluodu. Note: Non-Veg (Non-vegetation), Other-Veg (Other vegetation), *C. Dac (Cynodon dactylon), A. Ses (Alternanthera sessilis), C. Bon (Conyza bonariensis), Ch. Amb (Chenopodium ambrosioides), C. Can (Conyza canadensis), D. Rep (Dichondra repens), H. Sib (Hydrocotyle sibthorpioides), V. Off (Verbena officinalis), X. Sib (Xanthium sibiricum)*. (Generated with eCognition Developer, and the URL is https://www.ecognition.com).

According to the vegetation classification in the WLFZ of each study area (Fig. 5 and Table 4), the vegetation coverage of the study areas of the Liyuan, Ahai, Ludila and Guanyinyan reservoir WLFZ were all less than 5%. The study area of Ludila was completey devoid of vegetation in the WLFZ. The coverage in Liyuan was only 0.02%, with mostly individual herbaceous plants sporadically distributed on the upper boundary of the WLFZ. In Ahai, *C. dactylon* grow concentratly in patches at the top of the WLFZ together with some other sparsely growing vegetation, with a coverage of 1.47%. The vegetation coverage of Guanyinyan was 3.21%, mainly distributed in the upper part of the WLFZ and expanding towards the middle. In this area, 30.39% of the vegetation was *X. sibiricum*, growing in large tracts as low seedlings; 21.03% was *A. sessilis* growing in patches, 10.87% was *C. dactylon* growing mainly on the upper boundary of the WLFZ, and 37.71% was a mixture of plants growing in clusters with only a few of each.

**Table 4:** Vegetation area, vegetation coverage and vegetation classification accuracy of WLFZ in each study area.

Study area	Category		Area (m^2^)		Percentage		User accuray	Producer accuracy
Liyuan	Non-Veg		3630.10		99.98%		98.45%	98.22%
	Vegetation		0.59		0.02%		93.46%	93.46%
Ahai	Non-Veg		13,365.17		98.53%		98.02%	97.97%
	Vegetation	C. Dac	169.67	199.43	85.08%	1.47%	95.76%	93.34%
		Other-Veg	29.76		14.92%		97.55%	92.46%
Longkaikou	Non-Veg		7579.97		53.53%		97.21%	92.56%
	Vegetation	A. Ses	1743.43	6579.60	26.50%	46.47%	95.01%	90.62%
		C. Dac	4367.78		66.38%		93.31%	96.46%
		H. Sib	154.55		2.35%		92.03%	91.61%
		Other-Veg	313.84		4.77%		96.59%	92.83%
Ludila	Non-Veg		3724.76		100.00%		97.48%	98.20%
	Vegetation		0		0.00%		–	–
Guanyinyan	Non-Veg		7153.45		96.79%		98.21%	98.40%
	Vegetation	C. Dac	25.82	237.44	10.87%	3.21%	94.24%	87.80%
		X. Sib	72.15		30.39%		97.55%	98.33%
		A. Ses	49.92		21.03%		94.83%	97.65%
		Other-Veg	89.55		37.71%		98.17%	97.95%
Xiluodu	Non-Veg		2900.17		44.19%		98.25%	95.49%
	Vegetation	C. Dac	1026.93	3662.16	28.04%	55.81%	95.61%	96.41%
		X. Sib	2138.45		58.40%		94.38%	92.87%
		S. Vir	387.99		10.59%		96.52%	95.20%
		Other-Veg	108.79		2.97%		94.94%	97.25%

The vegetation coverage of Longkaikou and Xiluodu WLFZ was more abundant, 46.47% and 55.81% respectively. In Longkaikou, vegetation mainly covered the middle and upper parts of the WLFZ. Of the vegetation, 66.38% was *C. dactylon*, 26.50% was *A. sessilis*, 2.35% was *H. sibthorpioides,* 1.68% was *Ch. ambrosioides*, and 3.09% was a variety of vegetation species, only a few of each, divided into Other-Veg class.

Due to weather and equipment constraints, we were unable to photograph the upper and lower boundaries of the WLFZ in Xiluodu study area, but we still obtained the images of the main part of the WLFZ, which consisted mainly of 58.4% *X. sibiricum*, 28.04% *C. dactylon*, 10.59% *S. viridis*, and 2.97% other vegetation.

The vegetation coverage in the WLFZ of different reservoirs of the Jinsha River basin varied significantly, but in terms of quantity, most of them were absolutely dominated by 1–4 species, which were distributed in patches and strips, and covered an area and proportion far more than the rest of the vegetation, while the rest of the vegetation was sparse in quantity each and was sporadically distributed. *C. dactylon, A. sessilis, X. sibiricum, S. viridis, H. sibthorpioides, Ch. Ambrosioides* were the main dominant and pioneer species for vegetation restoration in the reservoir WLFZ of the Jinsha River basin.

### Spatial distribution pattern of vegetation in fluctuating zone

Since no vegetation survived in the Ludila study area, and the vegetation in the Liyuan, Ahai and Guanyinyan study areas was sparse, with less than 5% coverage, and all of them were concentrated in the upper part of the WLFZs (Fig. 5), this paper mainly analyzed the spatial distribution pattern of vegetation in the Longkou and Xiluodu study areas, which had better vegetation coverage.

#### Landscape pattern

CA is a basic index for landscape pattern study, and LPI reflects the proportion of the largest patch in the landscape type to the total landscape area, which is an expression of patch dominance. The SHAPE and PAFRAC describe the complexity of patch shape, the larger the SHAPE value indicates the more complex patch shape; the closer the PAFRAC value to 1 indicates the more regular patch shape. PROX reflects the degree of proximity of each landscape type, the larger its value indicates the higher degree of patch aggregation and the lower degree of fragmentation; ENN describes the degree of physical connection of the landscape types, the larger its value indicates the greater distance between patches and the greater degree of fragmentation.

**Figure 6:**
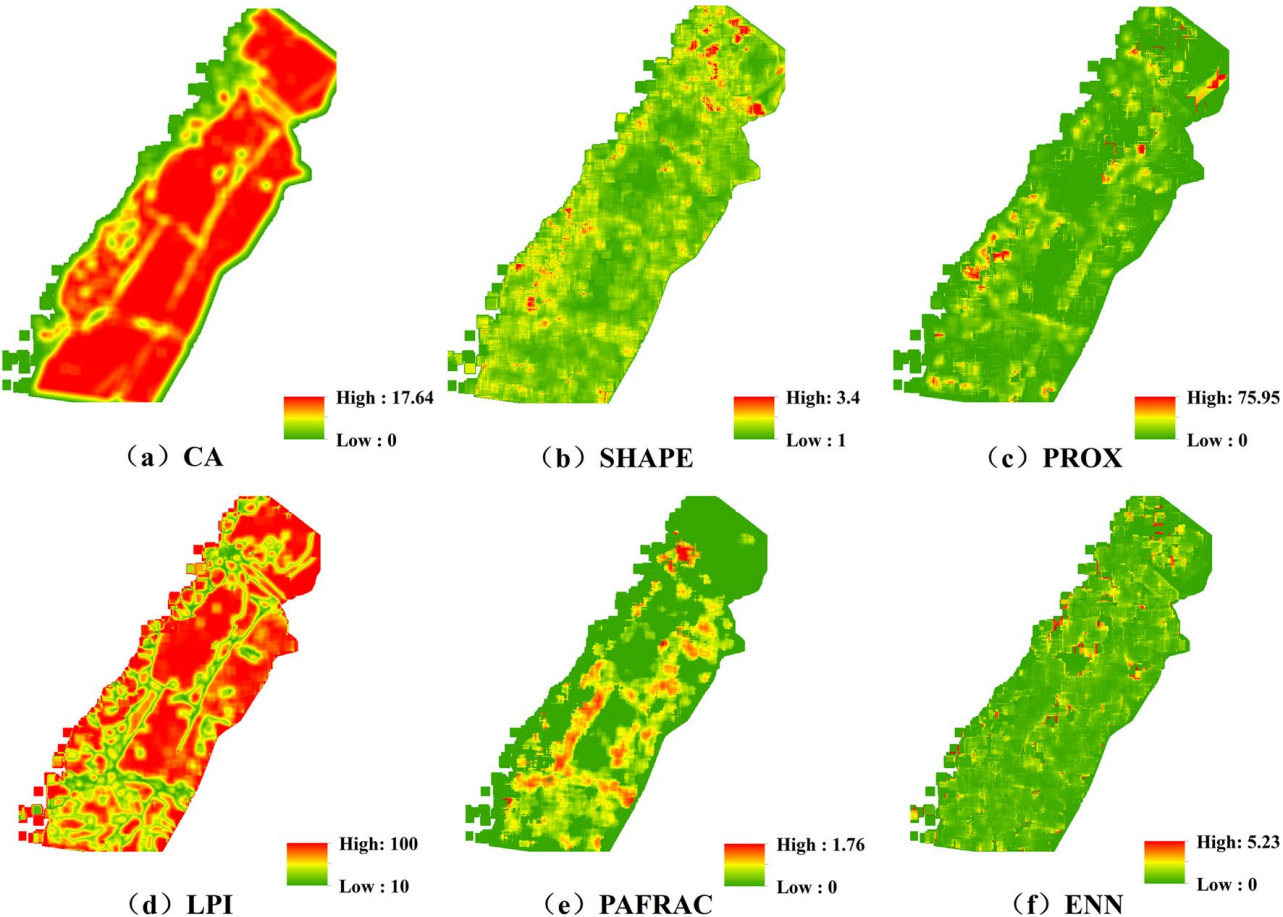
Spatial characteristics of vegetation landscape pattern index in the Longkaikou study area (Generated with ArcGIS 10.5 software, and the URL is: https://www.esri.com/en-us/home).

From the overall landscape level (Fig. 6), in the Longkaikou study area, CA and LPI showed that the areas of vegetation patches were large, less spatially fluctuating and uniform distribution, with obvious patch dominance, reflecting characteristics of patchy distribution; PROX and ENN showed that the vegetation patches were clustered and the landscape was well connected; SHAPE and PAFRAC showed that there was little variation in the shape complexity of vegetation patches in most areas of the WLFZ.

At the level of landscape types (Table 5), the vegetation landscape types in the Longkou study area included C*. dactylon*, *A. sessilis*, *H. sibthorpioides* and other vegetation, among which, *C. dactylon* showed significant advantages in patch area, patch dominance, patch aggregation and connectivity; followed by *A. sessilis* and *H. sibthorpioides*, *A. sessilis* was significantly better than *H. sibthorpioides* in patch area, but in patch shape, *H. sibthorpioides* was more aggregated than *A. sessilis* and had better patch connectivity; Other-Veg showed significant weaknesses in patch area and aggregation; there were no significant differences among the landscape types in patch shape.

**Table 5:** Landscape index of patch types in the Longkaikou study area.

Type	Area indicator		Shape indicator		Aggregate indicator	
	CA	LPI	SHAPE	PAFRAC	PROX	ENN
*C. dactylon*	4367.78	38.97	1.31	1.54	3599.78	0.65
*A. sessilis*	1743.43	4.24	1.41	1.26	89.47	0.75
*H. sibthorpioides*	154.55	1.28	1.58	1.48	106.62	0.72
Other-Veg	313.84	0.33	1.11	1.52	3.89	0.64

**Figure 7:**
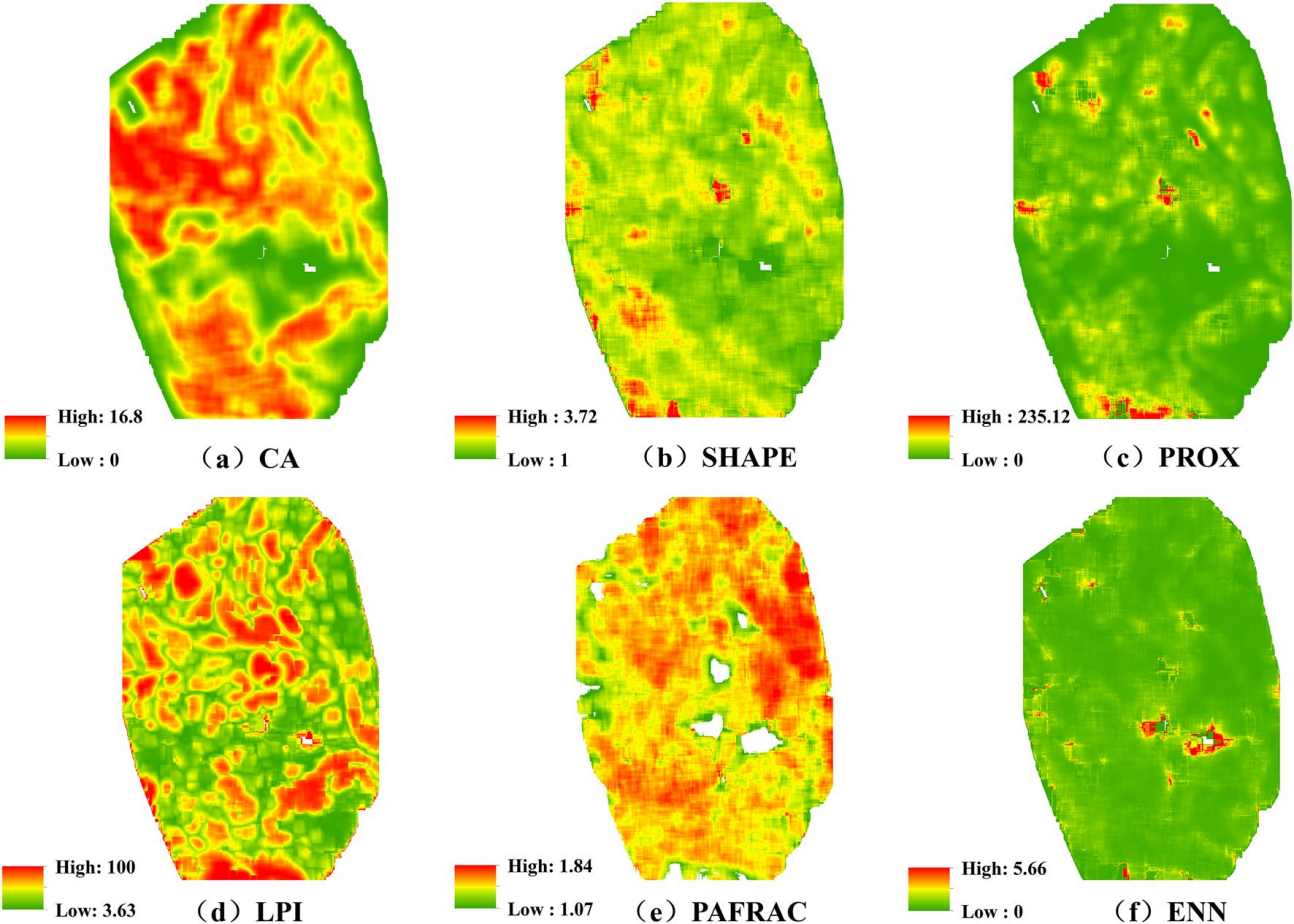
Spatial characteristics of vegetation landscape pattern index in the Xiluodu study area (Generated with ArcGIS 10.5 software, and the URL is:https://www.esri.com/en-us/home).

The spatial characteristics of the vegetation landscape pattern index in the Xiluodu study area were shown in Fig. 7. From the overall level of the landscape, the area of vegetation patches and the dominance of patches were spatially variable, the vegetation was well connected, with obvious characteristics of patchy distribution, and the shape of vegetation patches did not show obvious spatial characteristics.

From the level of landscape types (Table 6), the vegetation landscape types in Xiluodu study area included four categories: *X. sibiricum*, *C. dactylon*, *S. viridis* and Other-Veg type. Among them, *X. sibiricum* showed obvious advantages in patch area, patch dominance, patch aggregation and connectivity, followed by *C. dactylon*, both of which were significantly better than *S. viridis* and Other-Veg, and the differences in patch shape complexity among landscape types were small.

**Table 6:** Landscape index of patch types in the Xiluodu study area.

Type	Area indicator		shape indicator		Aggregate indicator	
	CA	LPI	SHAPE	PAFRAC	PROX	ENN
*X. sibiricum*	2138.45	9.64	1.37	1.49	955.55	0.27
*C. dactylon*	1026.93	7.78	1.26	1.54	291.05	0.27
*S. viridis*	387.99	0.53	1.17	1.58	9.34	0.29
Other-Veg	108.79	0.05	1.16	1.55	3.54	0.30

#### Distribution characteristics along terrain

**Figure 8:**
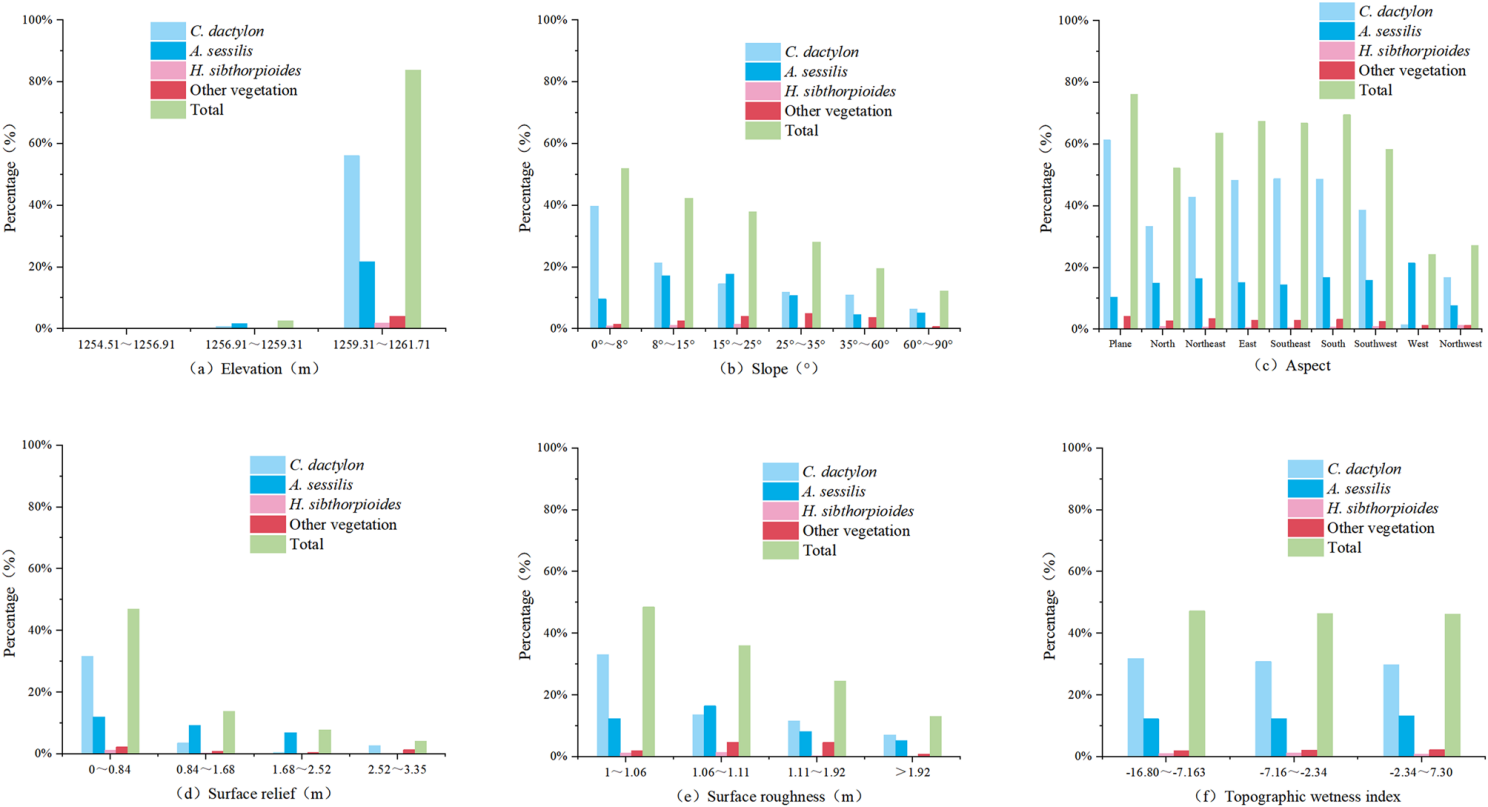
Changes in vegetation coverage with topographic factors in the Longkaikou study area (Drawn with Origin 2018_64Bit, and the URL is https://www.OriginLab.cn/).

According to the statistics (Fig. 8), the vegetation area share of Longkaikou study area in the upper, middle and lower elevation gradients of the WLFZ was 54.61%, 26.62% and 18.77%, respectively, indicating that the vegetation was mostly in the upper part of the WLFZ, with a coverage of 83.80%, while the vegetation in the lower part was the least, with a coverage of less than 1%. From the viewpoint of each vegetation species, in the upper part of the WLFZ, *C. dactylon* had the largest area, accounting for 66.9% of the total vegetation area, followed by *A. sessilis*, accounting for 25.9%, while *H. sibthorpioides* and Other-Veg only survived in the upper part, accounting for 2.3% and 4.9% each. From the distribution of each slope class, the vegetation of the WLFZ gradually decreased with the increase of slope, and the vegetation was mainly concentrated in the range of slope 35°, and the coverage of each vegetation decreased significantly when the slope exceeded 35°. In the aspect, the distribution of vegetation in the WLFZ did not show any obvious preference. The surface relief in the study area of Longkou was generally low, and *C. dactylon* was mainly distributed in the range of surface relief less than 0.84 m. When the surface relief is greater than 2.52 m, the vegetation coverage tends to be close to 0. The vegetation showed no obvious distribution preference in terms of surface roughness and topographic wetness index.

**Figure 9:**
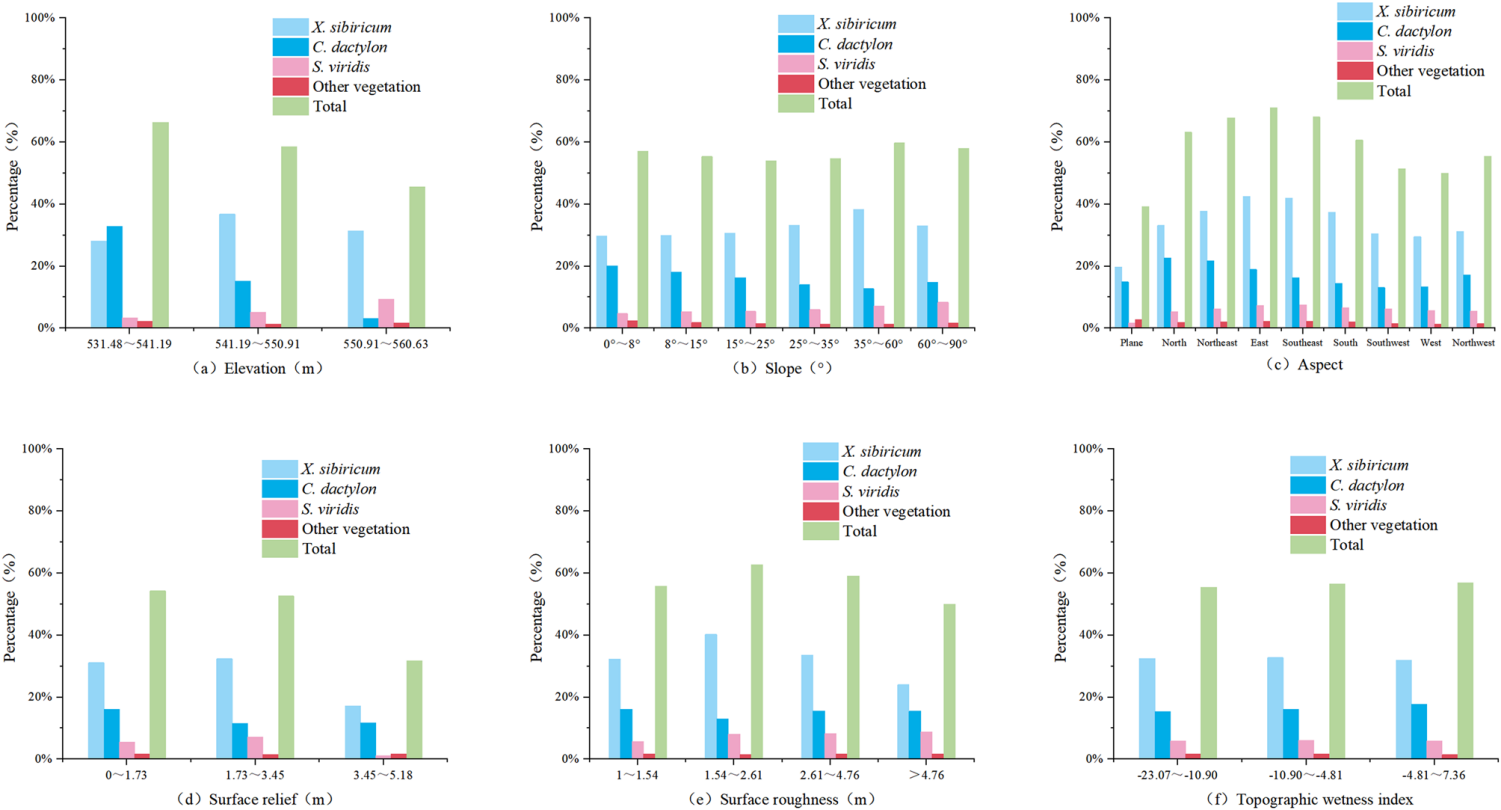
Changes in vegetation coverage with topographic factors in the Xiluodu study area (Drawn with Origin 2018_64Bit, and the URL is https://www.OriginLab.cn/).

The spatial distribution of vegetation in the study area of Xiluodu was shown in Fig. 9. The maximum drop in water level at Xiluodu study area can reach 60 m, but only the half of the upper part of the subsidence zone with a drop of about 30 m was photographed. The coverage rate of *C. dactylon* was the largest in this elevation gradient, *S. viridis* was mainly distributed in the uppermost part of the zone, while *X. strumarium* was well covered in all elevation gradients. From the distribution of surface relief, the overall vegetation coverage decreases with the increase of surface relief, with *X. strumarium* and *S. viridis* mainly distributed in the area of 0–3.45 m, while both the coverage of *C. dactylon* and Other-Veg were not much different across the surface relief . The distribution of vegetation showed no obvious preference in terms of slope, aspect, surface roughness and topographic wetness index.

### Influence of topographic factors on the spatial distribution pattern of vegetation in the WLFZ

**Figure 10:**
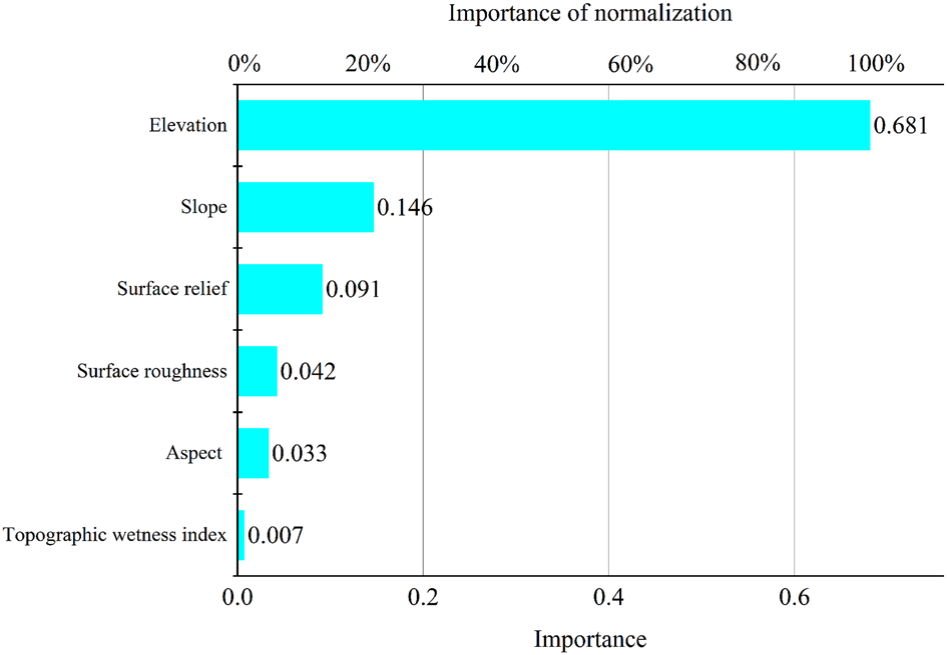
Ranking of important values of topographic factors in the Longkaikou study area (Drawn with Origin 2018_64Bit, and the URL is https://www.OriginLab.cn/).

According to the results of species distribution modeling, the number of samples in the study area of Longkaikou was 39,321, and the overall accuracy of the model was 88.2%. The terrain factors, in descending order of importance, were elevation > slope > surface relief > surface roughness > aspect > topographic wetness index, with values of 0.681, 0.146, 0.091, 0.042, 0.033 and 0.007, respectively (Fig. 10). It can be seen that the vegetation distribution in the WLFZ was mainly influenced by elevation, followed by slope and surface relief, and is less influenced by surface roughness, aspect and topographic wetness index. This was consistent with the results of typical correlation analysis.

A total of six pairs of typical variables were calculated in the Longkou study area, and standardized typical coefficients were used due to the inconsistency of each landscape pattern index as well as topographic factor units. According to the results of significance test (Table 7), the first four pairs of typical p-values were less than 0.05, indicating that the correlations reached a significant level, and their correlation coefficients were 0.565, 0.262, 0.142, and 0.034, among which the correlation coefficient of the first pair was the largest, so the first pair was selected for analysis. The topographic factors and landscape indices highly correlated with the first pair of typical variables were elevation, surface relief and CA and SHAPE, respectively. According to Tables 8 and 9, their mechanism of action was that the greater the elevation, the smaller the surface relief, resulting in a larger patch size and more complex shape of the vegetation, and therefore a more frequent exchange of energy with the outside world and a greater ability to survive.

**Table 7:** Significance test of typical correlation coefficient in the Longkaikou study area.

Number	Correlation	Eigenvalue	Wilks statistic	F	Num D.F	Denom D.F	Sig
1	0.565	0.469	0.620	255.869	36.000	80,218.776	0.000
2	0.262	0.073	0.911	68.668	25.000	67,864.110	0.000
3	0.142	0.021	0.978	25.079	16.000	55,813.355	0.000
4	0.034	0.001	0.999	2.989	9.000	44,464.530	0.001
5	0.017	0.000	1.000	1.399	4.000	36,542.000	0.231
6	0.005	0.000	1.000	0.411	1.000	18,272.000	0.521

**Table 8:** Standardized canonical correlation coefficients of terrain factors in the Longkaikou study area.

Variable	1	2	3	4	5	6
Elevation	− 0.626	− 0.749	− 0.233	− 0.055	0.004	0.024
Slope	0.230	− 0.503	1.188	0.213	− 0.197	0.147
Aspect	0.018	− 0.077	− 0.269	0.697	− 0.573	− 0.354
Surface roughness	− 0.116	0.235	− 0.459	− 0.685	− 0.128	− 0.891
Surface relief	0.737	− 0.299	− 0.702	− 0.121	0.261	0.223
Topographic wetness index	− 0.022	− 0.054	0.063	0.409	0.768	− 0.486

**Table 9:** Standardized typical correlation coefficients of landscape pattern in the Longkaikou study area.

Variable	1	2	3	4	5	6
CA	− 0.720	0.098	0.693	− 0.325	− 0.080	0.028
LPI	− 0.338	0.112	− 0.919	− 0.307	− 0.225	0.434
SHAPE	0.348	0.029	− 0.009	− 0.995	− 0.177	− 0.047
PAFRAC	− 0.123	-0.914	− 0.332	− 0.214	− 0.424	0.226
PROX	0.004	0.280	0.227	0.310	− 0.732	0.601
ENN	− 0.008	0.015	− 0.207	0.012	− 0.745	− 0.691

**Figure 11:**
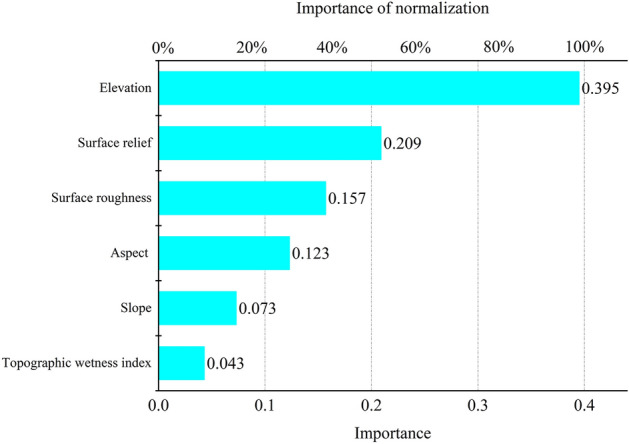
Ranking of important values of topographic factors in the Xiluodu study area (Drawn with Origin 2018_64Bit, and the URL is https://www.OriginLab.cn/).

The number of samples in the study area of Xiluodu was 41,010, and the overall accuracy of the model was 61.4%. The terrain factors, in descending order of importance, were elevation > surface relief > ground roughness > aspect > slope > terrain moisture index, with values of 0.395, 0.209, 0.157, 0.123, 0.073, and 0.043, respectively (Fig. 11). It can be seen that the vegetation distribution in the WLFZ was most influenced by the elevation, followed by the surface relief.According to the typical correlation analysis, six pairs of typical variables were calculated for the Xiluodu study area, of which the first four pairs had typical P values less than 0.05 (Table 10), indicating that the correlation reached a significant level, and their correlation coefficients were 0.299, 0.208, 0.102, and 0.033, and the first pair was the largest, so the first pair was selected for analysis.The topographic factors and landscape indices with high correlation with the first pair of typical variables were elevation,surface relief and CA, PAFRAC, respectively, and according to Tables 11 and 12, their mechanism of action was that the greater the elevation, the greater the surface relief, leading to a smaller patch area and simpler shape of the vegetation.

**Table 10:** Significance test of typical correlation coefficient in the Xiluodu study area.

No	Correlation	Eigenvalue	Wilks statistic	F	Num D.F	Denom D.F	Sig
1	0.299	0.098	0.861	173.177	36.000	180,037.588	0.000
2	0.208	0.045	0.946	92.320	25.000	152,306.027	0.000
3	0.102	0.010	0.988	29.793	16.000	125,257.707	0.000
4	0.033	0.001	0.999	5.556	9.000	99,785.811	0.000
5	0.010	0.000	1.000	0.992	4.000	82,004.000	0.410
6	0.001	0.000	1.000	0.027	1.000	41,003.000	0.869

**Table 11:** Standardized canonical correlation coefficients of terrain factors in the Xiluodu study area.

Variable	1	2	3	4	5	6
Elevation	− 0.728	0.602	− 0.327	0.013	0.046	− 0.002
Slope	− 0.021	− 0.570	− 0.926	− 0.441	0.061	0.034
Aspect	− 0.015	0.129	0.405	− 0.766	0.428	− 0.246
Surface roughness	0.049	0.140	0.324	0.665	0.842	− 0.149
Surface relief	− 0.681	− 0.503	0.538	0.155	− 0.206	− 0.030
Topographic wetness index	− 0.051	0.007	0.132	− 0.142	0.245	0.949

**Table 12:** Standardized typical correlation coefficients of landscape pattern in the Xiluodu study area.

Variable	1	2	3	4	5	6
CA	0.667	− 1.111	0.218	0.062	0.563	0.415
LPI	− 0.225	0.544	− 0.983	− 0.356	0.369	− 0.356
SHAPE	0.014	− 0.085	0.434	− 0.232	− 0.063	− 1.291
PAFRAC	− 0.613	− 0.195	− 0.549	− 0.301	− 0.683	0.681
PROX	0.532	0.430	− 0.095	0.214	− 0.994	0.317
ENN	0.279	0.020	0.328	− 0.792	− 0.020	0.670

### Limiting factors of vegetation restoration in WLFZ

**Figure 12:**
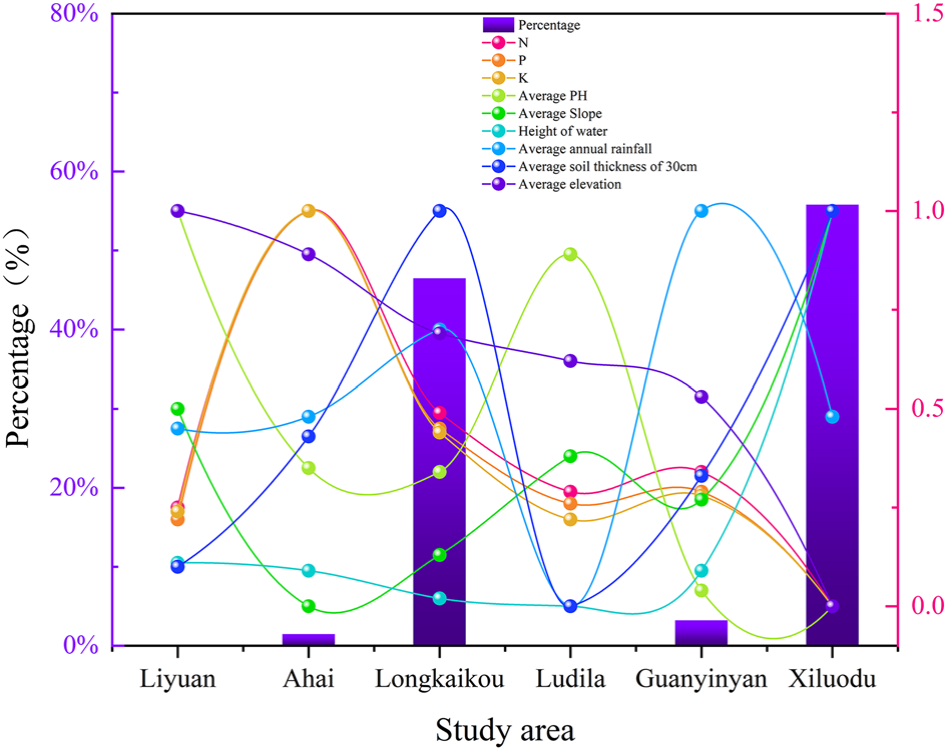
Statistical analysis of vegetation coverage and environmental parameters in each study area of Jinsha River Basin (Drawn with Origin 2018_64Bit, and the URL is https://www.OriginLab.cn/).

Preliminary studies showed that after long-term water level fluctuations in the cascade reservoirs, most of the vegetation in the WLFZs of the cascade reservoirs in the Jinsha River basin could be restored to different degrees, however, the restored species types were relatively simple, all of them were herbaceous plants, and mainly annual herbaceous plants. The restoration of the WLFZs of different reservoirs varied significantly, with vegetation coverage of more than 46% and 27 species types in the better restored areas, such as the Longkou study area, while the vegetation coverage of the less restored areas was usually less than 5% and 5–12 species types, and some areas even had no grass, such as the Ludila study area. According to the statistics (Fig. 12), the habitats in the study area of different reservoirs in the Jinsha River basin were significantly heterogeneous, with significant differences in climate, soil conditions, topography, and water level drop, etc. Because of the inconsistent range of values and units of different environmental factors, comparative analysis was performed by normalization, as shown in Fig. 12, vegetation cover was significantly correlated with the average soil Ph and the average thickness of the subsurface 30 cm soil layer, and the two study areas with average soil Ph greater than 8, Pear Garden and Rudyra, were almost completely bare. These two study areas were almost dominated by sand and gravel, with thin soils averaging < 5 cm in thickness. The lack of soil and difficulties in water retention exacerbated the aridity effect of climate and were not conducive to rapid plant establishment and growth, thus showing that soil substrates with Ph > 8 and soil thickness < 5 cm severely limit the recovery of vegetation in the WLFZ.

It was also noteworthy that, according to Fig. 12, the elevation of each study area did not seem to show a significant correlation with its vegetation cover. However, the analysis in “Influence of topographic factors on the spatial distribution pattern of vegetation in the WLFZ” section specifically focused on topographic factors showed that elevation was the main factor affecting vegetation restoration. In fact, the two were not contradictory, because compared with climate and soil factors, elevation was an indirect environmental factor. When conducting statistical analysis of different reservoir study areas in the whole Jinsha River Basin, elevation mainly reflected the differences of climate and photothermal conditions among the study areas.when microtopography of the same study area was analyzed, elevation mainly reflected the inundation stress and drought stress suffered by different elevation gradients of the subsidence zone in the study area, etc. Generally, the flooding time and depth in the lower part of the WLFZ were sequentially greater than those in the middle and upper part, while the outcropping time and drought stress in the upper part are sequentially greater than those in the middle and lower part. According to the vegetation classification maps (Fig. 5), the vegetation in the study areas mainly survived in the upper and middle parts of the WLFZs, while the lower vegetation coverage was less, especially in the lower part of the riverside area, almost no vegetation survived, which means that in the WLFZs of the arid valley, flooding stress has a more obvious restriction on vegetation survival than drought stress. The lower part of the WLFZs endures an average of 8–11 months of complete deep flooding every year, which makes most plants difficult to survive.

## Discussion

### Effects of reservoir water level regulation on species composition in the WLFZ

The Jinsha River arid valley is often the preferred site for hydropower development due to its unique alpine canyon topography. The study areas of liyuan, Ahai, Ludila, Guanyinyan and Xiluodu reservoir WLFZ are located in the middle and lower reaches of the Jinsha River arid valley section. From the relevant studies^49–54^, before the construction of the reservoirs, the vegetation types of the arid river valley section on both sides of Jinsha River below 1600 m above sea level was generally "sparse trees- shrubs- grasses", i.e., scattered trees and shrubs on top of large grassland vegetation, and the community structure was mostly divided into three layers of trees, shrubs and grasses or two layers of shrubs and grasses. In terms of plant species composition, most of them were tropical (or tropical origin) drought-tolerant species, characterized by small-leaved, stiff-leaved and thorny vegetation, among which herbaceous dominant species include *Heteropogon contortus, Bothriochloa pertusa,* etc., shrub dominant species include *Dodonaea viscosa, Vitex negundo,* etc., and tree dominant species include *Phyllanthus emblica, Bombax ceiba,* etc.

In this survey, only 44 species of vascular plants were found in the WLFZs of the Jinsha River arid valley reservoirs, all annual or perennial herbs, and no trees and shrubs were found. Among them, a large number of the original species before the construction of the reservoirs^55–57^ have died out*,* with only *O. corniculata*, *B. pilosa*, *S. orientalis*, *C. rotundus*, *C. bonariensis*, *S. viridis* and *V. officinalis* surviving. It indicated that after the reservoir filling, the flooding stress made most of the original plants unable to adapt and were eliminated, and the few species that could adapt survived. This provided an opportunity for invasive alien species. Of the species found in this survey, 66% were invasive plants^58–61^ and 61% were also reported in the Three Gorges Reservoir WLFZs, where the dominant species, *C. dactylon, X. sibiricum* and *A. sessilis,* had good flood tolerance and were also common dominant species in the Three Gorges Reservoir WLFZs. The Jinsha River dry valley reservoirs and the Three Gorges reservoir have their own different climatic, soil, topographic and other native site conditions, but the difference between them in the existing species composition of the WLFZs were relatively small, which reflected that water level variation is the dominant factor influencing the vegetation species composition in the WLFZ, and may show convergence in the natural selection process of suitable biological species in the WLFZ.

After the impoundment of cascade reservoirs in Jinsha River, great changes in water level, anti-seasonal hydrological conditions and long-term winter flooding had a strong filtering effect on plants in the fluctuating zone. The species composition was simplified, and the existing plants were mainly herbaceous. Of these, the number of annuals amounted to54.55%, able to complete their life history during the outcrop period in the WLFZ, relying on seed dormancy to avoid prolonged winter flooding; perennial herbs amount to 34.09%, which can cope with inundation stress through their strong clonal capacity^62^, and tree shrubs, which have almost disappeared. Vegetation restoration in the Jinsha River reservoir WLFZs was still in its initial stage. The existing species composition and dominant species types were similar to those of the Three Gorges Reservoir. However, it was worth noting that 17 endemic species have been found in the Jinsha River reservoir WLFZs , which have not been reported in other reservoirs. Whether these endemic species will compete to win and take over the position of existing dominant species over time will require longer-term locational monitoring. These fixed observation plots established in the WLFZs of reservoirs in the Jinsha River Basin can lay the foundation for subsequent long-term observation. Under the interference of repeated flooding, the natural reconstruction of vegetation in the WLFZ is a long-term and complex process, which requires longer continuous observation to reveal the internal mechanism of vegetation community construction and evolution in the WLFZ of reservoir.

### Discussion of ecological restoration strategies in the WLFZ

The quality of the vegetation in the WLFZ is an important indicator of the health of the reservoir and plays an important role in the river ecosystem. At present, a series of vegetation restoration studies and practices have been carried out for some large reservoir WLFZs, the main difficulties were that, the substrate for vegetation growth in the fluctuating zone was easily lost by water scouring, and the basic conditions for plant growth were constantly destroyed; the ecological environment of the WLFZ was harsh, with plants having to endure long periods of deep water inundation and drought stress during the dry period, especially in the dry river valley WLFZ, where the water-heat conflict was more pronounced; there were very few plant species in nature that can adapt to the alternating wet and dry environment of the reservoir WLFZs.

At present, the ecological restoration of the WLFZ was carried out in two main ways: the selection of suitable plants and the construction of suitable habitats. In the selection of suitable plants, many researchers have conducted numerous studies on the community structure of vegetation in the reservoir WLFZs and tested the submergence tolerance of plants through a large number of flooding simulation tests, and screened out some suitable flood-tolerant species for the WLFZ, including *Morus alba, Betula nigra, Taxodium ascendens, Taxodium 'Zhongshanshan', Taxodium distichum, Salix variegata, C. dactylon* and *Chrysopogon zizanioides* etc. The planting pattern was usually based on planting herbaceous species in the lower part of the WLFZ, followed by shrub species as the elevation increases, and flood-tolerant tree species near the highest water level in the upper part of the WLFZ. However, long-term monitoring has shown that the survival rate of trees and shrubs is low, for example, all Morus alba planted in the WLFZ of the Three Gorges Reservoir at elevations 140-169 m died, the survival rate at elevations 170-171 m was 7.8%, and only the survival rate in the range of 3 m below the highest water level of 175 m can be high in the form of dwarf shrubs, but it is degrading year by year^24,63^. So far, there are rare reports of plants that can survive after their canopies have been submerged for 3–6 months. Vegetation restoration trails in the WLFZ of Jinping reservoir in the Yalong River also showed that trees and shrubs barely survived, while herbaceous plants only recoverd in the upper part of the gently sloping WLFZ, where inundation lasted less than five months. This also implied that shortening the inundation time may promote the recovery of vegetation in the WLFZ. Reservoir scheduling was usually formulated according to the changing pattern of incoming runoff, taking into account power generation, flood control, sand control, navigation, irrigation, etc^64,65^. In recent years, the idea of ecological scheduling of rivers has been proposed^66,67^, which mainly refers to taking the ecological needs of benthic organisms into account while ensuring the original objectives of the reservoir. However, the ecological needs of the WLFZ were rarely considered at present. At this point, reservoir ecological scheduling still needs to be further explored, taking into account not only benthic organisms but also, as far as possible, the ecological needs of the WLFZ, for example in terms of the duration and depth of inundation tolerance of plants. This required cross-fertilisation across fields and an in-depth integration of hydrological scheduling research with the study of WLFZ plants.

In the construction of suitable habitats in the WLFZ, the plants were usually provided with the necessary standing conditions by engineering measures in the early stages of their growth, and the soil matrix was reinforced by plant roots bridging the gap between the engineering measures and the slope bank in the later stages. At present, the most widely used methods include vegetated concrete slope protection greening techniques and thick base material slope protection greening techniques, etc. In addition, some adaptive strategies of water level change have been put forward to provide a stable water environment for vegetation by storing water in the fluctuating area, so that vegetation can better adapt to the change of water level. Such methods include swallow's nest planting holes, layered water storage strategies, base pond systems and nine-grid greening planting tray techniques, etc.

The special habitat of the WLFZs of the Jinsha River dry valley reservoirs have received less research related to vegetation restoration due to short formation time and complex geographical environment. The first-hand survey of the area's WLFZs in this paper showed that all extant species were annual or perennial herbs, as shown in Table 2, and could be considered as alternative species for restoration, but no trees and shrubs were found.

Restoration practices in existing reservoirs, such as the Three Gorges Reservoir, have shown that the survival rate of trees and shrubs was low, with survival only occurring near the highest water level of the WLFZ, where the inundation was shallowest and the inundation time was shortest. However, the drought stress was relatively more intense near the highest water level in the WLFZ of dry river valley reservoirs. There is great uncertainty as to whether tree species selected from existing reservoir WLFZ restoration trials, such as the Three Gorges Reservoir, can be adapted to the dry river valley WLFZ. At present, vegetation restoration in dry river valleys mainly focused on tree planting and introduction of exotic species, ignoring the dominant role of native plants. The use of tree species to directly establish top communities has proven to be ineffective, and most of the vegetation restoration patterns dominated by trees experienced extensive decline, especially exotic species, which were often poorly adapted and severely divided^51,53,54^. Therefore, attempts to introduce tree and shrub species screened from other reservoir WLFZs, such as the Three Gorges Reservoir, into arid river valley WLFZs were not optimistic. For example, *Betula nigra, Taxodium ascendens* and *Taxodium distichum* were physiologically water-loving and flood-tolerant, and whether they can adapt to the dry river valley climate needs further testing. In contrast, native plants have undergone long-term natural selection, and their morphological structures were adapted to the special climatic and soil conditions of arid river valleys, which have greater ecological adaptability compared with exotic species and were conducive to the stability and sustainable development of the ecosystem. Therefore, vegetation restoration should be adapted to local conditions and pay attention to native species.

Since the reservoirs in Jinsha River were successively built and stored in water, the original vegetation in the WLFZs has been flooded and died out, forming a large area of secondary bare land. The natural restoration of vegetation is a long process, and it is difficult to form vegetation cover in a short time. With the continuous erosion of water, the surface soil is gradually lost, the soil layer becomes thinner and even the bedrock is exposed, the foundation on which plants depend is lost and revegetationwill be more difficult, as in the case of the Ludila and Liyuan study areas. The vegetation restoration of the WLFZ should take the prevention of soil erosion as the first priority, targeting the three main limiting factors of soil foundation, flooding stress and drought stress, screening flood and drought tolerant plants from native species, grasses as appropriate, promoting the establishment of the initial ecosystem of the WLFZ through artificial planting, accelerating the formation of vegetation cover, and preserving the soil foundation for subsequent sustainable vegetation recovery and succession, and following up mainly by natural restoration without deliberate intervention, as more and more practices proved that it was almost impossible to directly reconstruct the top community through artificial intervention.

The spatial distribution pattern of vegetation in the WLFZ was significantly influenced by elevation, surface relief and slope. The natural recovery of vegetation in the Jinsha River basin reservoir WLFZs generally followed an elevation gradient from the upper part of the WLFZ downwards, so manual interventions can also be considered in the upper part of the WLFZ and gradually move downwards along the elevation gradient. In particular, for areas such as the Liyuan and Ludila study areas, where sand and gravel were dominant and the soil was thin, the first step should be to create substrate conditions for plant growth, and reference can be made to existing bioengineering models such as the Three Gorges Reservoir WLFZ, where suitable habitats can be constructed through engineering measures, such as the configuration of guest soil vegetation along contour strips or patches, gradually progressing from top to bottom along the elevation gradient, is recommended.

Surface relief and slope affected the change of surface runoff and the water and soil retention capacity of the WLFZ. With the increase of surface relief, the vegetation cover gradually decreased, the fragmentation of vegetation landscape pattern increased, and the complexity of vegetation patch shape decreased. The fragmentation of landscape patterns often led to population segregation, and the simple shape of patches was not conducive to material-energy exchange between plants and the outside world, which together increased the risk of extinction of local populations due to random disturbance, which to a certain extent explained the decrease in plant cover when surface relief increases. For patches with complex shapes, there were more edge areas, which were easy to carry out material circulation and energy flow with the outside world. The edge effect of patches affected the size and survival rate of plant patches by changing the ecological environment and influencing ecological processes. Usually, the surface relief was greater than 3 m, the slope was greater than 35°, the soil was easy to be washed off, and the vegetation growth conditions were harsher. In this regard, it was suggested that certain bioengineering measures should be supplemented to slow down soil erosion and supplement the matrix conditions for plant growth to promote plant colonization. While for the gentle slope with small topographic relief, natural restoration and bioengineering measures can be used to promote the rapid formation of vegetation cover in the initial stage, and natural restoration was the main method in the subsequent stage.

### UAV remote sensing helps to study the vegetation in the reservoir WLFZ

Reservoir dams were often built in steep, high mountain valley areas with complex terrain, and traditional ground surveys^68^ were often inaccessible in many places, so that only part of the measurable data at the sampling points can be obtained and the coverage was small.Remote sensing was capable of obtaining continuous observations over large areas in a non-contact manner, regardless of terrain.As most of the vegetation in the WLFZ was low herbaceous plants, according to the remote sensing information extraction test in this paper, it was only possible to distinguish the dominant species types at the centimetre level of spatial resolution. Therefore, the spatial resolution of satellite remote sensing and aerial remote sensing often cannot meet the needs of fine identification of vegetation in the WLFZ, and both of them were easily obscured by cloud cover.UAV remote sensing was mobile and flexible, capable of flying at low altitude under clouds and obtaining a high enough spatial resolution at a suitable flight altitude to clearly identify vegetation types, which was ideal for vegetation surveys in reservoir WLFZ with complex topography, as demonstrated by the research in this paper.

**Figure 13:**
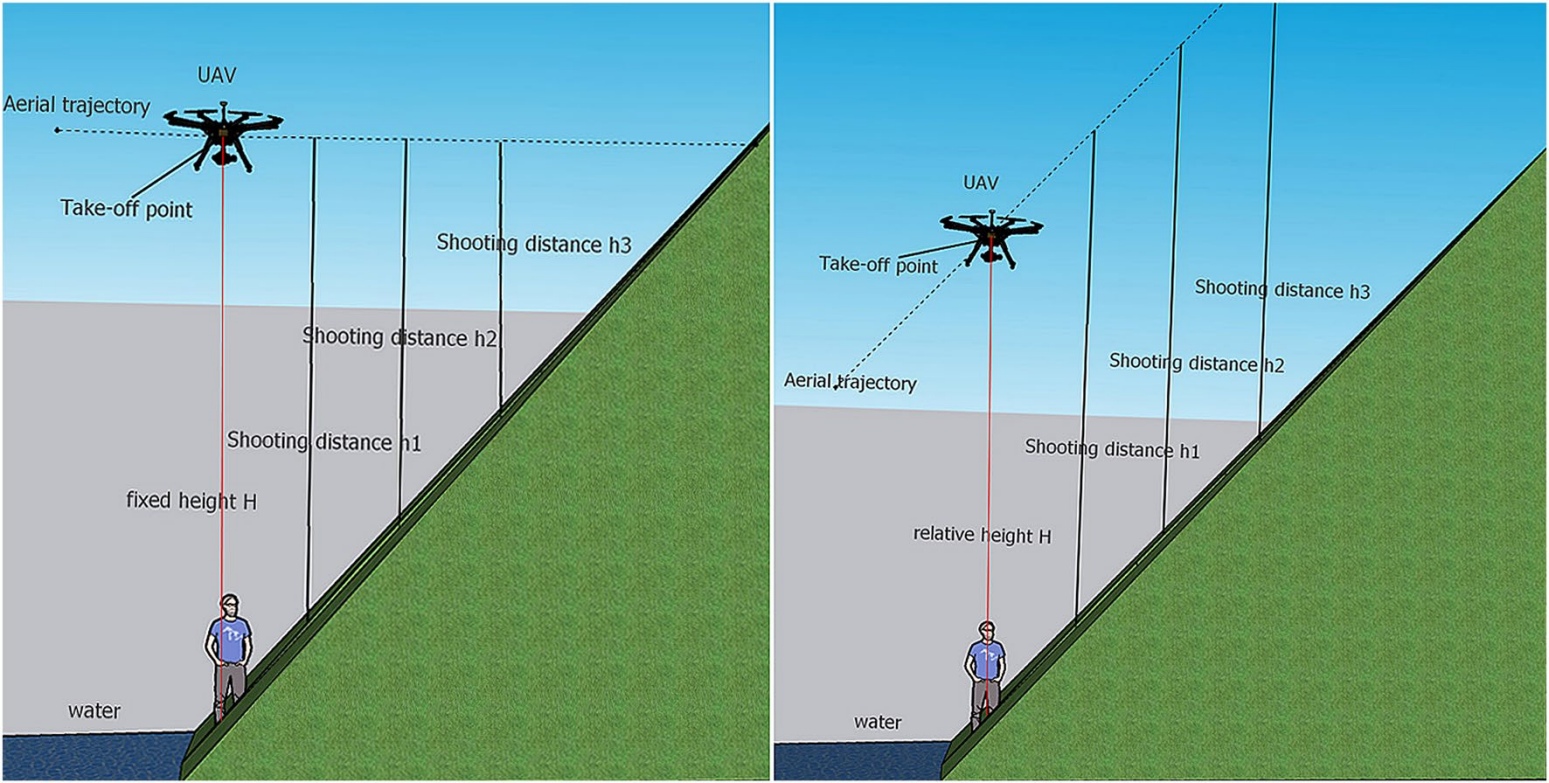
Schematic diagram of fixed altitude and relative altitude flight (Drawn with SketchUp 2020, and the URL is https://www.sketchup.com).

Usually the UAV flight control software provided two flight modes, automatic route planning and manual flight. In automatic route planning, once the flight altitude h was set, it will keep flying in the horizontal plane with vertical height h above the take-off point (Fig. 13). However, the topography of the reservoir WLFZ was usually undulating, for example, the height difference of the Xiluodu reservoir WLFZ can be up to 60 m, which will lead to a large difference in the shooting distance in different areas of the WLFZ, thus resulting in a large difference in the spatial resolution of the shooting, which was not conducive to the subsequent quantitative extraction of vegetation. Therefore, manual flight was usually adopted to facilitate flexible adjustment of flight altitude to cope with the inconsistent shooting distance caused by the change of terrain undulation. However, when observing and judging the height of aerial photography at different locations, UAV flyers inevitably suffered from visual bias, especially when flying over large distances, where the human eye has a large bias in observing the flight height. It was also difficult to judge and control the degree of overlap in the side and heading in manual flight, which relies heavily on the experience and operational skills of the flyer. In this regard, it was recommended that the UAV and the corresponding flight control software can develop a relative flight height mode (Fig. 13), automatically judge and adjust the shooting distance in real time during the flight. This was not only for the WLFZ, but also for other areas with undulating terrain, the relative altitude mode will greatly enhance the convenience and safety of field operations.

## Conclusions

After the construction of the reservoirs, the original drought-tolerant "sparse trees- shrubs- grasses" in the reservoir WLFZs of the Jinsha River arid valley evolved into amphibious annuals and perennial herbaceous plants, and the trees and shrubs have disappeared. Reservoir water level changes showed convergence in the natural screening process of suitable species in the WLFZ. The vegetation species composition of the WLFZs of the Jinsha River Arid Valley Reservoirs and the Three Gorges Reservoir, both of which use "winter storage and summer discharge" water level scheduling, is less different, despite their very different climates, soils and topographies. The vegetation in the WLFZ showed obvious band-like aggregated distribution characteristics along the water level elevation gradient, and was mainly concentrated in the upper part of the WLFZ, dominated by patches of *Cynodon dactylon*, *Xanthium strumarium*, *Alternanthera sessilis*, *Setaria viridis* and *Hydrocotyle sibthorpioides*, with almost no vegetation growing in the lower near-water area. Even in arid river valley WLFZ, flood stress exhibited a more pronounced limiting effect on vegetation survival relative to drought stress. In addition, the lack of soil substrate was also a major limiting factor for vegetation restoration in the WLFZ. Steep slopes with large topographic relief were relatively more susceptible to scouring, difficulties in water and soil retention, and high rates of exposed bedrock, which were not conducive to vegetation growth. The restoration of vegetation in the WLFZ should be based on prevention first, with natural ecological restoration as the main focus and biological engineering as a supplement. At the initial stage of secondary bare land restoration, priority should be given to the upper part of the WLFZ with less topographic relief for artificial planting trials, gradually advancing downwards along the water level elevation gradient, while the areas of the WLFZ with slopes greater than 35° and larger topographic relief should be supplemented with bioengineering measures to help plant colonisation, and after a certain amount of stable cover has been formed, natural restoration should be the main focus. The restoration plants were recommended to be selected from the existing native species obtained from the research, with emphasis on the dominant role of native species and grasses where appropriate, as practices have shown that it was almost impossible to introduce exotic species to directly establish a top community.

## Data Availability

The public data such as DEM data, can be downloaded directly through the link provided in Fig. 1. However, other data that support the findings of this study are all confidential data in China. According to the requirements of relevant laws, these confidential data have been decrypted when we use them. Any researchers in related fields that need these decrypted data can contact the corresponding author to obtain them.
